# Phenotypic evolution from genetic polymorphisms in a radial network architecture

**DOI:** 10.1186/1741-7007-5-50

**Published:** 2007-11-14

**Authors:** Arnaud Le Rouzic, Paul B Siegel, Örjan Carlborg

**Affiliations:** 1 Linnaeus Centre for Bioinformatics, Uppsala University, Box 598, SE-75124 Uppsala, Sweden; 2 Centre for Ecological and Evolutionary Synthesis, Department of Biology, PO 1066 Blindern, 0316 Oslo, Norway; 3 Virginia Polytechnic Institute and State University, Department of Animal and Poultry Sciences, Blacksburg, VA 24061-0306, USA; 4 Department of Animal Breeding and Genetics, Swedish University of Agricultural Sciences, Box 7023, SE-750 07 Uppsala, Sweden

## Abstract

**Background:**

The genetic architecture of a quantitative trait influences the phenotypic response to natural or artificial selection. One of the main objectives of genetic mapping studies is to identify the genetic factors underlying complex traits and understand how they contribute to phenotypic expression. Presently, we are good at identifying and locating individual loci with large effects, but there is a void in describing more complex genetic architectures. Although large networks of connected genes have been reported, there is an almost complete lack of information on how polymorphisms in these networks contribute to phenotypic variation and change. To date, most of our understanding comes from theoretical, model-based studies, and it remains difficult to assess how realistic their conclusions are as   they lack empirical support.

**Results:**

A previous study provided evidence that nearly half of the difference in eight-week body weight between two divergently selected lines of chickens was a result of four loci organized in a 'radial' network (one central locus interacting with three 'radial' loci that, in turn, only interacted with the central locus). Here, we study the relationship between phenotypic change and genetic polymorphism in this empirically detected network. We use a model-free approach to study, through individual-based simulations, the dynamic properties of this polymorphic and epistatic genetic architecture. The study provides new insights to how epistasis can modify the selection response, buffer and reveal effects of major loci leading to a progressive release of genetic variation. We also illustrate the difficulty of predicting genetic architecture from observed selection response, and discuss mechanisms that might lead to misleading conclusions on underlying genetic architectures from quantitative trait locus (QTL) experiments in selected populations.

**Conclusion:**

Considering both molecular (QTL) and phenotypic (selection response) data, as suggested in this work, provides additional insights into the genetic mechanisms involved in the response to selection. Such dissection of genetic architectures and in-depth studies of their ability to contribute to short- or long-term selection response represents an important step towards a better understanding of the genetic bases of complex traits and, consequently, of the evolutionary properties of populations.

## Background

For decades, evolutionary and quantitative geneticists have suspected that genetic interactions are important for phenotypic evolution. In numerous empirical studies, genetic interactions (epistasis) have implied important contributions to differences among and within species for various quantitative traits, such as plant morphology and growth [1-3], animal behavior and physiology [4-6], metabolism [7] or fitness [8-14] (see [15-18] for reviews). It is now fairly well acknowledged that biological systems rely on complex networks of interacting genes (e.g. [19-21]); however, the extent to which genetic polymorphism in these networks contribute to phenotypic variation remains to be shown.

There is an almost complete lack of insight on mechanistic contributions of genetic interactions to phenotypic change. Thus, it is important to identify such mechanisms empirically and utilize them as realistic starting points for in-depth theoretical explorations of the importance of genetic architecture on phenotypic evolution. For instance, an expectation from theoretical models is that certain forms of epistatic interactions may promote sustained long-term responses to selection, reaching an ending point that is much higher than what could be predicted from the observable genetic variation in the initial population (e.g. [22-24]). Recent support for this idea comes from studies [25,26] that reported that strong interactions were important in two of the most famous long-term artificial selection experiments: the century long Illinois corn selection for oil and protein concentration [27] and the 50-generation body weight selection experiment in the Virginia chicken lines [28].

**Figure 1:**
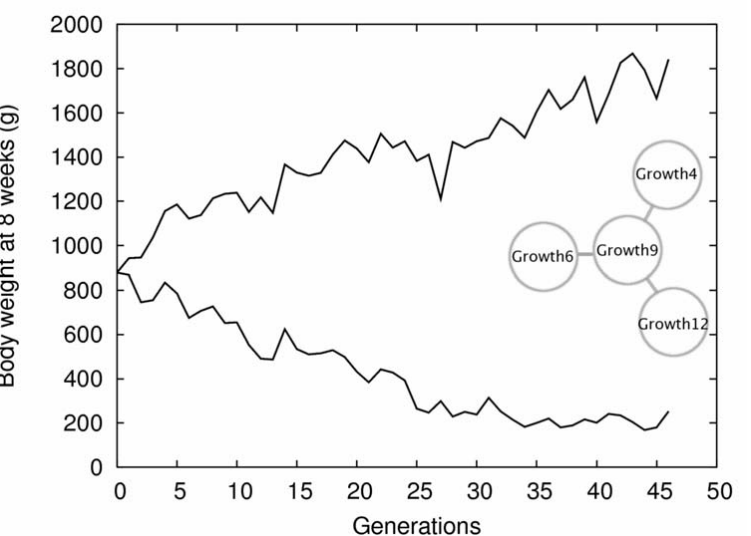
**Observed response to selection**. Average male body weights are plotted for high (top curve) and low (bottom curve) selected lines [33]. After 46 generations of directional selection, the lines differ by around 1600 g. The response to selection in the low line reached a plateau after about 30 generations, probably because of physiological constraints. The four-loci genetic network evidenced in [26] has been represented on the right.

The availability of high-quality datasets with good estimates of the phenotypes corresponding to each genotypic combination of a genetic architecture of interest (genotype-phenotype map) facilitates explorations of the link between genetic interactions and phenotypic evolution, by providing functional estimates of epistasis independent from allelic frequencies in the population [29,30]. Data from an intercross between the Virginia high and low selected lines used by Carlborg et al [26] provided a unique example of a biological system where strong genetic interactions were involved in the phenotypic change of a quantitative character under strong artificial selection (Figure 1). A network of four interacting loci explained nearly half of the eight-fold body weight difference between the chicken lines, and these data provided direct estimates for the phenotypes corresponding to any genotypic combination among these four loci. In this paper, we use individual-based computer simulations to explore the dynamic properties of this gene network when subjected to divergent phenotypic selection resembling that of the original selection experiment. The results from the study are used to highlight the impact of radial genetic network architectures on phenotypic evolution and lead to a discussion of the impact of epistasis on the possibility to dissect the genetic architecture of complex traits.

## Results

### Genetic modeling and network architecture

In traditional statistical genetic models, epistasis describes the multiple locus genetic effects that cannot be explained by additivity and dominance [31]. However, these statistical interaction effects are only valid in the population where they have been estimated. Therefore, they are not useful for inferring unambiguous biological relationships between loci or their potential contribution to the selection response for a population over time. A more useful tool for studying responses to selection from genetic data is the genotype-phenotype map, i.e. a high-quality dataset associating a phenotypic value to each genotype. In this paper we use the dataset from which Carlborg et al [26] observed strong epistasis affecting body weight (see Methods). In the four-locus genotype-phenotype map, alleles inherited from the high line (H) and the low line (L) are combined to form (theoretically) 81 diploid genotypic combinations in the F_2 _population. The data allowed direct estimation of phenotypic means for 77 of these 81 genotypes.

**Figure 2:**
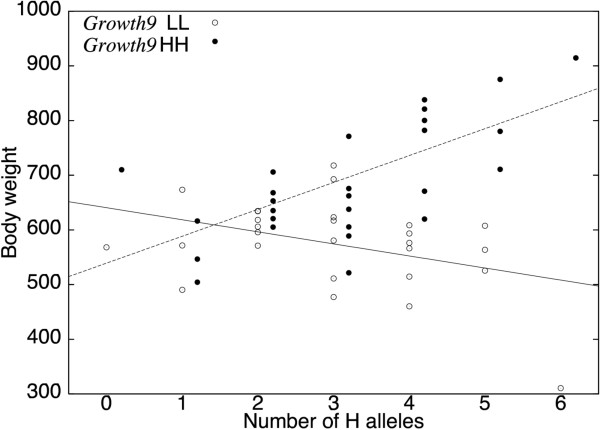
**Effect of the number of H alleles at the radial loci (*Growth4*, *Growth6*, *Growth12*) depending on the genetic background at *Growth9 *(HH or LL)**. Each of the dots represent the average of a genotypic combination, lines illustrate the corresponding regressions in each of the *Growth9 *backgrounds. For clarity, *Growth9 *HL genotypes are not plotted (in this background, H alleles have a slight positive effect, roughly intermediate between the effects in HH and LL backgrounds).

Further analysis shows that the effect of the central locus of the network (labeled *Growth9*, according to [26]) is very different from the effects of the three other 'radial' loci (*Growth4*, *Growth6 *and *Growth12*). Figure 2 illustrates how H alleles at the three radial loci increase or decrease the phenotype depending on the genetic background at the central locus *Growth9*. In homozygous *Growth9 *HH individuals, H alleles lead to a heavier body weight, while these same H alleles decrease body weight in a *Growth9 *LL genetic background. Each of the radial loci thus reveals (or buffers) the genetic effect of *Growth9*. In the studied F_2 _population with intermediate allelic frequencies at all loci, only part of the maximum effects of the network can be described using marginal additive effects. This explains why *Growth9 *was the only genome-wide significant quantitative trait locus (QTL) in a standard analysis [32]: on average, the three radial loci had much smaller additive effects and, hence, could not be detected in a one-dimensional QTL analysis.

### Genetic network and response to selection

The evolutionary properties of the four-locus genotype-phenotype map were explored using computer simulations. Each individual in the simulated population is represented by its genotype and by its phenotype, calculated as the sum of the genotypic value from the genotype-phenotype map and a random environmental effect (see Methods). Artificial populations were submitted *in silico *to arbitrary selection pressures, designed to mimic the artificial selection performed in the original selection experiment to generate the high and low body weight selected chicken lines.

#### Highly variable response to selection

**Figure 3:**
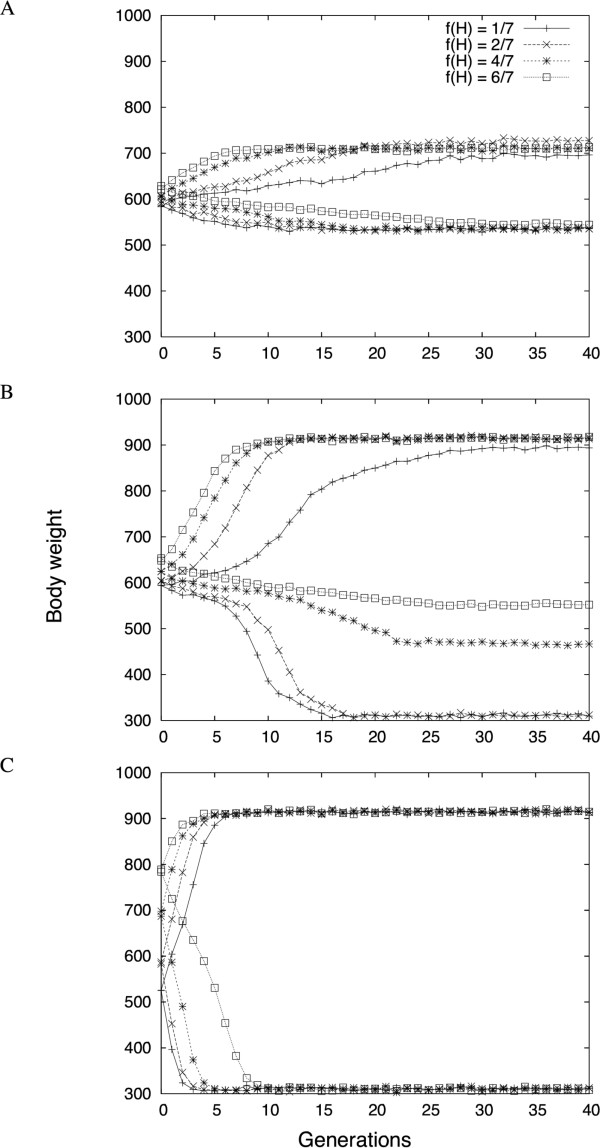
**Expected dynamics (average over 10 simulations) in high and low lines with different initial H allele frequencies for the central and radial loci**. To simplify the analysis, initial frequencies at radial loci have been considered as identical. The radial loci H frequency is (A) 1/7, (B) 3/7 and (C) 6/7. In each part, the phenotypic response to sselection is plotted for different H allele   frequencies at the central locus (1/7, 2/7, 4/7, 6/7, see Methods).

The rate of phenotypic change, as well as the final outcome of the selection process, depends on the initial H-allele frequency *f*_0_(H) (Figure 3). As the radial loci display a similar effect in their interactions with *Growth9*, they have been assigned the same initial allelic frequency. We found several divergent dynamics, which differ depending on the initial allelic frequencies of the central and/or radial components of the network. An intermediate *f*_0_(H) leads to symmetrical responses during bi-directional selection [33]. Low H frequencies in the radial loci (Figure 3A) correspond to a buffered (canalized) system on which selection is not efficient: the response to selection is very slow, and the system can be trapped in local sub-optimal states. In contrast, decanalization owing to H alleles at the radial loci (Figure 3C) leads to very rapid selection responses. The dynamics that lead to the most symmetric and progressive selection responses occur with intermediate initial frequencies in both central and radial loci (Figure 3B).

#### Response to directional selection

**Figure 4:**
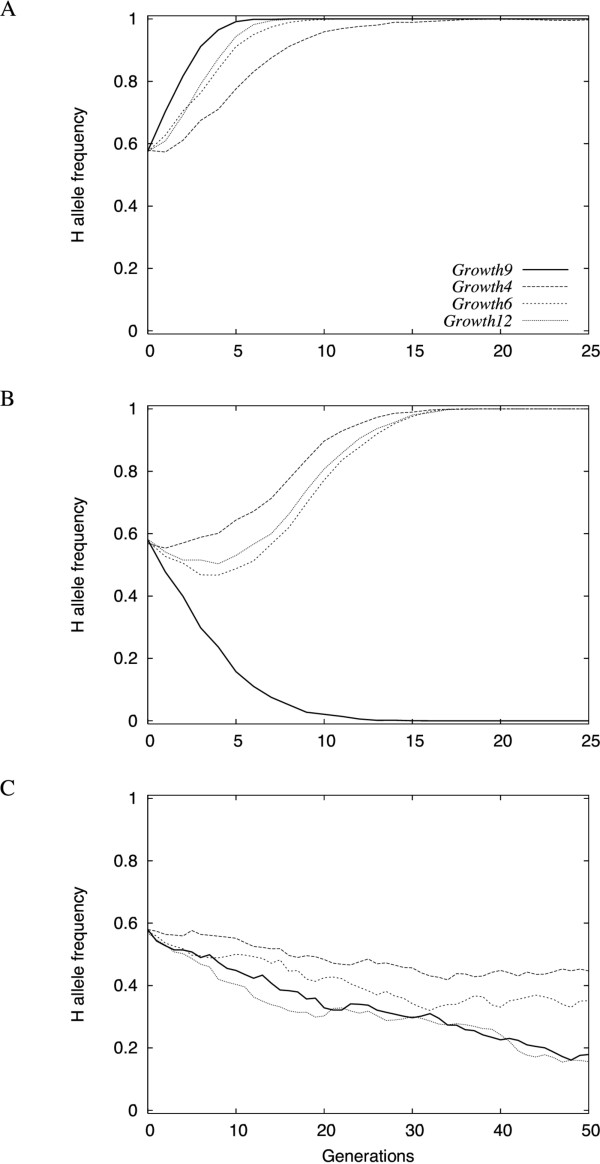
**Dynamics of allelic frequencies under different selection pressures (averages over 10 simulations)**. Plain line: central locus (*Growth9*); dotted lines: radial loci (*Growth4*, *Growth6 *and *Growth12*). Initial frequencies are set to *f*(H) = 4/7 for all loci, which seems to be one of the most likely situations (see Figure 3). (A) Upwards directional selection; (B) downwards directional selection; (C) downwards directional selection including a decrease of the fitness for low-weight individuals, simulating physiological constraints: the normal truncation selection is performed, and then the fitness of low-weight individuals is modified (if the weight is lower than 450 g, then the fitness is 0, and fitness increases linearly between 450 and 550 g; above 550 g, the fitness is maximal and set to 1). In this last situation, the selection intensity has been decreased (40 individuals randomly drawn among the 200 best phenotypes) to avoid too much genetic drift (selection in both sides sometimes lead to much less than 40 fertile adults with the 'standard' selection strength).

The dynamics of *f*(H) were studied for each of the four loci in the network under upward and downward directional selection (Figure 4A and 4B). At any point in the genotype-phenotype map, the central locus in the network (*Growth9*) displays the largest individual additive effect with the same sign in all genetic backgrounds. As a result, *Growth9 *is systematically fixed first, for H and L alleles in the high and low lines, respectively, in all evaluated scenarios. The effects on the phenotype (either high or low) from the radial loci (*Growth4*, *Growth6 *and *Growth12*) are gradually revealed by increases in *Growth9 f*(H) and as a result they are fixed after *Growth9*. Under directional selection, *Growth4*, *Growth6 *and *Growth12 *are fixed for H alleles in both the high and low lines. This result is rather surprising, given that, by definition, L alleles were called 'L' owing to their origin in the low line.

**Figure 5:**
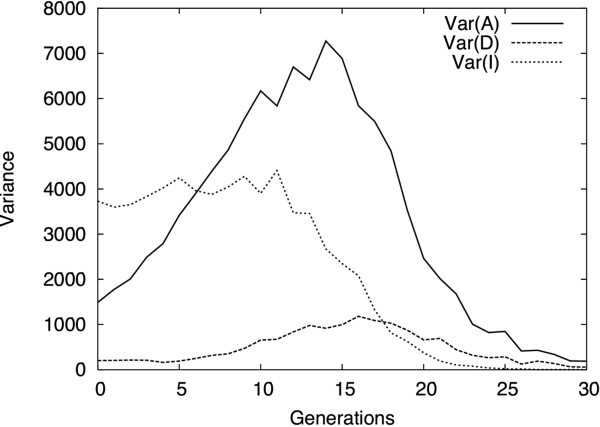
**Dynamics of the variance components**. The data correspond to the average variances computed over 10 simulations of the same high-line selection experiment (initial frequencies of H alleles: 3/7): Var(A), additive variance; Var(D), dominance variance; Var(I), interaction variance, including any kind of second- or higher-order epistasis, where Additive × Additive epistasis is the major component (not shown). The initial population, with intermediate frequencies, is close to the F_2 _population, with a low additive variance and a high epistatic variance. With the changes in allelic frequencies owing to the selection response, the epistatic variance component slowly decreases while the additive variance (and heritability) increases up to a maximum (between generations 10 and 15 under these conditions), which also corresponds to the maximum speed of the selection response (see Figure 3). The dominance component remains very low during the entire process, having a negligible impact on the overall dynamics.

According to standard quantitative genetics theory, genetic variance consists of additive, dominance and interaction components [34]. The additive genetic variance describes the main resemblance between parents and offspring and is thus the best predictor of the immediate selection response in a population. Figure 5 shows the dynamics of these genetic variance components in the simulated populations selected for increased phenotypes, illustrating how interaction (epistatic) variance present in the initial population is progressively turned into additive variance. This induction of additive genetic variance sustains the selection response much farther than could be expected given the small amount of additive genetic variation available at the initiation of the selection experiment. This clearly illustrates how dependent the amount of additive genetic variance is on the allelic frequencies in a selected population and also shows the advantage of modeling epistatic effects when aiming at addressing the evolutionary properties of genetic architectures.

#### Stabilizing selection

A general observation in selection experiments for decreased body weight is that the response ceases after a number of generations owing to physiological constraints. In the Virginia lines, the response in the low line has been small since generation 30 (Figure 1). The resulting balance between natural and artificial selection resembles stabilizing selection with an intermediary optimum. In simulations with stabilizing selection in the low line, we show that this can lead to fixation of the L allele for all four loci in the network (Figure 4C). This illustrates the unconventional manner in which genetic network architectures influence phenotypic evolution and that this could make it difficult to detect such patterns in line-cross experiments between selected lines.

#### Linear responses to selection

**Figure 6:**
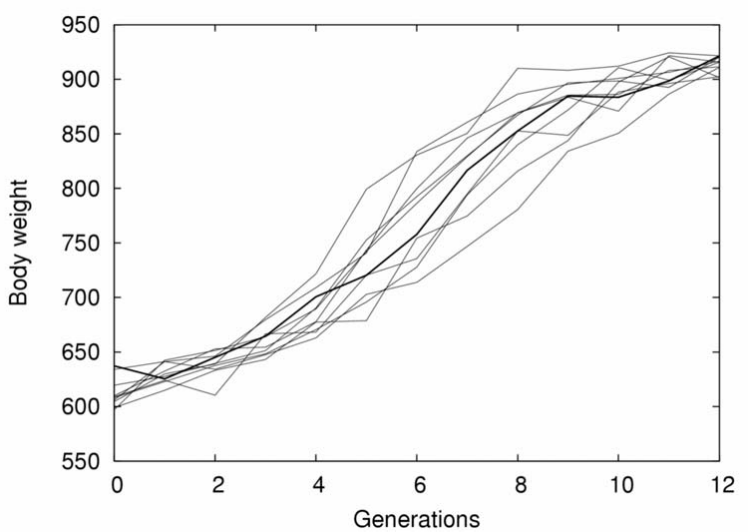
**Influence of genetic drift on the response to selection**. Strong positive (synergistic) epistasis is often supposed to lead to sigmoid responses to selection, with an acceleration of the selection response when favorable genotypic combinations appear in the population. This is also what can be expected for the genetic network described in this article (see, e.g., Figure 3B). However, this sigmoid shape was not observed during the experimental selection process (see Figure 1). Among other hypotheses, random genetic drift can easily hide this theoretical acceleration of the selection response. This figure details 10 simulations of the first generations of the selection response in the high line (same parameters as Figure 3B), and shows how, despite a clear overall sigmoid shape, some single simulations can easily appear to respond very regularly to selection (one of them is highlighted).

Many empirically observed selection responses where genetic interactions are implied, e.g. the Virginia chicken lines, the Illinois corn lines and others [35], are nearly linear (Figure 1). The expected response to selection from the radial four-locus network is, however, slightly sigmoid (Figure 3). In actual biological systems under selection, the response most likely results from a genetic architecture composed of a mixture of loci with marginal effects of varying size as well as loci with interaction effects. To explore how the contribution of other components of a more complex genetic architecture affect selection response, we made simulations where several minor genetic factors were included in addition to the four-locus epistatic network. This is a clearly realistic scenario as the four-locus networks contributes to half the selection response in the Virginia lines, and thus other genetic factors need to contribute as well. The result is that the selection response is increasingly linearized when the number of additional QTLs increases (see additional file 1: SupFig1). Moreover, because random fluctuations, most likely a result of genetic drift (given the relatively small population sizes generally implied in artificial selection procedures), observed responses from the four-locus network do not systematically exhibit the slightly sigmoid pattern. Simulations confirm that even if the average response to selection is not linear, individual realizations can range from linear to sigmoidal, with many being too noisy to show a clear pattern (Figure 6).

## Discussion and conclusion

### QTL-based approach of the population history

Artificial selection experiments generally provide dynamic data, such as heritability, changes in response to selection and estimates of environmental perturbations. Combining these empirical data with the results from a QTL analysis based on an intercross between selected lines is likely to enhance the understanding of the genetic architecture underlying the dynamical properties of the population. Unfortunately, several details of the selection will always remain unknown and thus preclude attempts to directly fit the output of the simulations with the observed selection response.

#### Explanatory power of detected genetic architectures

Advances in analytical methods for QTL detection has made it possible to unravel gene interaction networks affecting complex traits [15,26]. QTL studies will, however, fail to uncover genetic factors with small individual or interaction effects that segregate in the original lines or that display genetic inheritance patterns that do not conform to the genetic models used in the analysis (e.g. third-order epistasis or multi-allelic QTLs). Consequently, although the four-locus network detected in the Virginia chickens explains nearly half of the original difference between lines, other factors contributed about 75% of the phenotypic variability among the informative individuals in the F_2 _population. As illustrated in additional file 1: SupFig1, minor genetic factors of weak effects that cannot be detected (and, therefore, not included in the simulations) may modify the expected responses to selection significantly.

The genotype-phenotype map that is the foundation of our study may itself contain some statistical uncertainties. The estimates of genotypic values are based on a finite-size F_2 _population and will thus be affected by sampling effects, in particular for the completely homozygous genotypes. The sampling could have an impact, in particular, on the selection limit, but less of an effect on the overall dynamics (see additional file 2: SupFig2). Furthermore, as the loci were detected in a QTL experiment, their effect might be overestimated owing to the Beavis effect [36-38]. However, because the major focus in our study was on the dynamic properties of selection on a radial network genetic architecture, the negative impact of overestimated QTL effects should be minor, as these properties will hold even if the magnitude of the effects of the network was biased.

In a selection experiment, intrinsically random factors, such as genetic drift or environmental noise, are eventually likely to interfere with the theoretically expected selection response. Owing to randomness (genetic drift, sampling errors and perhaps also environmental intergeneration variation), it appears that the empirically measured phenotype sometimes evolves in the direction opposite to the selection pressure (in the Virginia lines, this happened 17 times over 46 years in the high line, and 12 times over the first 35 generations in the low line). These factors can be accounted for in simulations, and we used an effective population size resembling that used in the production of the Virginia lines, as well as an environmental effect based on the amplitude of the non-genetic effects measured in the F_2 _population. Theoretical results [23,39-42] predict that genetic drift in small or fragmented populations can have a large impact on the evolution of epistatic characters, and simulated replicates of the same selection pattern evidence the potential impact of genetic drift on the overall shape of the phenotypic evolution (Figure 6).

#### Benefits of combining QTL and empirical selection data

It is widely acknowledged that the link between the genetic architecture and responses to selection is not direct [35], and that inferences about genetic factors from the output of selection experiments remain largely speculative. However, considering both molecular (QTL) and phenotypic (selection response) data, as suggested in this work, provides additional insights into the genetic mechanisms involved in the response to selection of these lines of chickens, and more generally in long-term sustained selection responses.

The state of the initial population, which remains one of the main unknowns when parameterizing the simulations, can also be successfully addressed. We know that the base population of the original selection experiment was formed by the crossing of seven partly inbred chicken lines. There are, however, no empirical estimates of the initial frequencies of the H and L alleles in these lines. We have therefore explored the dynamics in the selection response for a large range of initial states of the base population. The results show that the expected dynamics of the network changes dramatically depending on the initial frequencies of alleles, from almost no phenotypic improvement (and a final state far from the theoretical optimal phenotype) to very fast (a few generations) fixation of the best genotype. None of these extreme dynamics correspond to the regular response to selection observed empirically during the artificial selection process, and can therefore be ruled out: the allelic frequencies of H and L alleles were probably roughly even in this particular initial population. This also suggests that, in a polymorphic genetic background, no allelic marginal effect seems to be sufficiently high to allow spread (from very low frequency to fixation) in the time scale of the selection experiment. It is thus unlikely that new mutations, i.e. alleles that were not present initially, would have had a major impact on the selection response in this particular case, and the initial population was probably already polymorphic for all loci of the network.

Furthermore, theoretical *in silico *explorations of the reconstructed gene network by submitting it to directional selection facilitates a better understanding of the respective roles of the different components of the genetic architecture. The simulations show that the effects of the major genes in the network were probably rather weak at the beginning of the selection experiment, and that responses during the very early generations were likely to be partly a result of minor genetic factors. In all simulations, the central locus was the first fixed, and its fixation enhanced the effects of the other loci, which then subsequently contributed to the selection response. Our results therefore suggest that the selection response is composed of different stages that lead to the observed linear phenotypic change when they overlap. Dissecting genetic architectures and identifying their ability to contribute in the short or long term to selection response may represent an important step towards a better understanding of the genetic bases of complex traits and, consequently, of the evolutionary properties of populations.

### Selection response from genetic networks

Genetic networks can be defined at any interaction level, from molecular to high-level complex phenotypes, such as, in our example, body weight. The evidenced gene network has been defined through significant epistatic interactions that were detected by a QTL analysis. One cannot deduce whether these four loci correspond to genes that are part of the same metabolic pathway or, on the contrary, participate in independent high-level functions (e.g. physiology, anatomy, behavior) involved in a complex way in the expression of body weight. In any case, the fact that each of the radial components of the network has a similar effect on the phenotype, both in nature and in amplitude, through similar interactions with a unique central component (as illustrated in Figure 2) remains particularly striking, and strongly supports the hypothesis of the biological meaning of this network.

Although QTL detection experiments are generally not powerful enough to detect networks as large as those deduced from metabolic studies [43], they can confirm that the complexity of a biological system is higher than the sum of its components (individual genes or individual pairwise interactions). As shown in this paper, an important difference in the realized response to selection from radial network architectures compared with selection responses from individual loci *per se *is the dependence on the initial allelic frequencies at other loci to achieve the full genetic potential for phenotypic change. Furthermore, as the effects of the network components are in the same direction, there are likely to reinforce each other and to lead to clearer patterns. For instance, the genetic network described in our study shows positive epistasis (i.e. synergistic epistasis) when selected towards heavier body weights, where the effects of the H alleles reinforce each other. Such directional epistasis is known to induce a different response to selection than that expected from the traditional additive model (e.g. [44-46]), and the theoretical resulting selection curve is a sigmoid (Figure 3), with a slow response in the first generations (changes in allelic frequencies without major consequences on the phenotype) followed by a 'burst' of additive variance when the favorable genotypic combinations become more frequent. It is not clear whether such an effect might have been detected (or even might have a significant effect on the phenotypic change) if only two components (e.g. the central locus and one of the radial factors) would have been involved.

### QTL detection of genetic networks

Studying crosses between divergently selected or evolutionary distant populations remains a powerful way to infer the genetic architecture underlying selection response to natural or artificial selection (e.g. [47]). One of the strongest assumptions in this approach is that the original populations are fixed for alternative alleles or at least segregating for multiple different alleles. If, however, the same allele is fixed in both populations owing to the underlying genetic architecture, one will fail to detect these loci.

A surprising, but intriguing, result in our study is that the genotype-phenotype map contains several genotypes that display lower phenotypes than the genotype of the selected 'low line'. The lowest weights are obtained for the genotype with LL homozygosity for the central locus and H alleles at the radial loci. The low line illustrates a scenario where the artificial selection experiment was disturbed by natural selection that eliminated extreme phenotypes: the smallest chickens in the low line were often unable to reproduce or even to survive until reproductive age [47]. This can be observed in Figure 1, because the response to selection ceases in the low line after approximately 30 generations despite a reasonable amount of remaining genetic variation. This unexpected selection pattern might explain why the L alleles fixed in the low line do not lead to the lowest phenotype; they rather lead to the optimal phenotype selected through stabilizing selection.

It is often assumed that the part of the genetic variability that cannot be detected in the experiment is a result of numerous minor QTLs, QTLs in a repulsion phase or epistasis. Minor effect QTLs and, to a lesser extent, epistasis could potentially be detected by increasing the quality of the data (i.e. more genotyped individuals and more markers). Here we indicate a genetic architecture that no line-cross-based QTL experiment, regardless of the accuracy of the statistical tools or the number of sampled individuals, would be able to evidence as the same allele is fixed in all lines; only the central locus will be found and implied to have a major additive effect. Indeed, if natural selection would have been bypassed in the experiment, then the final fixed genotype would likely have been the same in both high and low lines for *Growth4*, *Growth6 *and *Growth12 *and the conclusion from the QTL experiment would have been that a single QTL, *Growth9*, was the major factor of the selection response in both lines. Consequently, the complexity of the genetic architecture that has contributed to the phenotypic evolution would remain unknown and, furthermore, as the effect of this locus is entirely dependent on its context, this could be a problem in future efforts to clone the underlying gene or use it, for example, in marker-assisted selection.

## Methods

### Chicken lines

From a common base population, constituted by a mixture of seven partially inbred lines, two chicken lines have been selected for eight-week body weight; one towards high weights (high line, H) and the other towards low weights (low line, L). After 46 generations of artificial selection [33,47,48], the average weight of the high line (1412 g) was about eight times the average weight of the low line (170 g); see Figure 1. The lines were crossed and 795 individuals of the resulting F_2 _were genotyped for 145 genetic markers. A standard QTL analysis for eight-week body weight in the F_2 _population revealed only one genome-wide significant QTL located on chromosome 7 (*Growth9*). Three additional loci, *Growth4*, *Growth6 *and *Growth12 *(located on chromosomes 3, 4 and 20, respectively) were evidenced by a pairwise analysis [26], all of these three loci interacting significantly with *Growth9*. *Growth9 *emerges as the central point of a radial epistatic genetic network (Figure 1).

### F_2 _population and genotype-phenotype map

A subset of 538 individuals, which were the most genetically informative for the four loci in the genetic network with a large effect on growth [26], were used to design the genotype-phenotype map. In this report we refer to alleles from the low line and the high line as L and H, respectively. There are three possible genotypes (LL, HL or HH) at each locus, with twice as many heterozygous individuals as homozygous, owing to the F_2 _design. Among the 3^4 ^= 81 possible genotypes, we obtained information (i.e. an estimate of the average phenotype) for 77 genotypes, with between 1 and 38 phenotyped individuals per genotype. The total phenotypic variance in this dataset is σP2
 MathType@MTEF@5@5@+=feaafiart1ev1aaatCvAUfKttLearuWrP9MDH5MBPbIqV92AaeXatLxBI9gBaebbnrfifHhDYfgasaacPC6xNi=xH8viVGI8Gi=hEeeu0xXdbba9frFj0xb9qqpG0dXdb9aspeI8k8fiI+fsY=rqGqVepae9pg0db9vqaiVgFr0xfr=xfr=xc9adbaqaaeGacaGaaiaabeqaaeqabiWaaaGcbaacciGae83Wdm3aa0baaSqaaiabbcfaqbqaaiabikdaYaaaaaa@2FE3@ = 2.82 × 10^4 ^and the residual variance (i.e. taking into account both environmental effects and minor genetic factors) is σR2
 MathType@MTEF@5@5@+=feaafiart1ev1aaatCvAUfKttLearuWrP9MDH5MBPbIqV92AaeXatLxBI9gBaebbnrfifHhDYfgasaacPC6xNi=xH8viVGI8Gi=hEeeu0xXdbba9frFj0xb9qqpG0dXdb9aspeI8k8fiI+fsY=rqGqVepae9pg0db9vqaiVgFr0xfr=xfr=xc9adbaqaaeGacaGaaiaabeqaaeqabiWaaaGcbaacciGae83Wdm3aa0baaSqaaiabbkfasbqaaiabikdaYaaaaaa@2FE7@ = 2.13 × 10^4^. The four missing phenotypes were estimated from the mean of the closest genotypes, and simulations with different values have been run to confirm the minor impact of these estimates on the general dynamics of the system (see additional file 3: SupFig3).

### Simulations

Individual-based computer simulations were performed to explore the dynamics of the four-locus system when submitted to artificial selection. Each individual *i *was characterized by its genotype *g*_*i *_(i.e. the allelic combination carried at each of the four loci of the network) and by its phenotype *P*_*i*_. The artificial selection used relied on a truncation process. In each generation, a given number of individuals *N*_S _were allowed to reproduce depending on their phenotype (highest weight individuals in the high line, lowest weight individuals in the low line). For simplicity, the sex ratio was considered as even. For each of the *N*_T _individuals expected in the following generation (*N*_T_/2 males and *N*_T_/2 females), two parents (one male and one female) were randomly drawn. Each parent gives a 'gamete' (i.e. a haploid combination of their genotype, loci being considered as genetically independent), and both gametes were merged to constitute the genotype of the new individual *i*. The phenotype *P*_*i *_was then computed by *P*_*i *_= *G*_*i *_+ *ε*_*i*_, where *G*_*i *_is the phenotypic value of the genotype *g*_*i *_directly read from the genotype-phenotype map and *ε*_*i *_is a random factor following a normal distribution N(0, *σ*_R_), corresponding to environmental effects.

### Parameterization

One of the main unknowns of the system is the state of the initial population. The selection experiment, started in 1957, was initiated from a polymorphic population obtained by crossing seven partly inbred chicken lines (inbreeding coefficient around 50%). Unfortunately, no tissue or DNA samples exist from these lines and any initial allelic frequency is potentially realistic for each of the four loci, leading to many possible initial scenarios. However, for simplicity we considered that the polymorphism of the four loci in each of the seven initial inbred lines can be neglected and, thus, allelic frequencies at generation 0 were a multiple of 1/7.

The simulation of the selection procedure has been slightly simplified compared with the real selection. Population sizes have been considered as constant, with *N*_T _= 300, while the actual number of individuals fluctuated depending on the environmental and economical conditions (between 126 and 428, mean 269 in the high line, and between 83 and 511, mean 309 in the low line). Simulations evidenced that random fluctuations of the population size have no effect on the overall dynamics (see additional file 4: SupFig4).

The intensity of the selection process was considered as constant in simulations, while it was not the case during the actual selection procedure. During the four first generations, 8 males and 48 females were mated, and the intensity of selection has been slightly decreased to 12 males/48 females for generations 5 to 25, and 14 males/56 females thereafter. These correspond to effective population sizes of 27.4, 38.4 and 44.8 individuals, respectively, and we chose to simulate them by a constant *N*_S _= 40 parents at each generation. Preliminary simulations show that these simplifications only had a minor impact on the system (see additional file 4: SupFig4). Moreover, the actual selection intensity was lower than what can be expected from the proportion of selected individuals. There was concern about the potential loss of genetic diversity and to limit inbreeding: upper limits were placed on family sizes and there was avoidance in mating closely related chickens. This modified and less-stringent truncation selection was simulated by truncating 100 individuals and drawing the *N*_S _= 40 parents randomly among these 100 individuals.

Finally, the environmental variation was estimated from the F_2 _population, where the phenotypic variance that could not be explained by the four loci was *σ*_R_^2 ^= 2.13 × 10^4^. The value of *σ*_R _has thus been fixed at 146 g and includes both the environmental variance and the genetic variation that cannot be explained by the identified QTLs.

## Authors' contributions

The project was initiated by OC, and all authors participated in the design of simulations, discussion and interpretation of results. PBS and OC provided and analyzed phenotypic and molecular data, respectively. AL performed and analyzed the simulations. AL and OC wrote the manuscript. All authors provided comments on the manuscript.

## Supplementary Material

Additional file 1 Effect of the presence of extra low-effect QTLs on the expected dynamics. The average selection response of the network itself is compared with that of the network plus six small-effect independent loci. Increasing the number of small additive QTLs clearly leads to a more linear response that fits better with the experimental data. Click here for file

Additional file 2 Impact of the sampling effect. The genotype-phenotype map was evaluated from a finite F_2 _population and the estimates of genotypic values are necessarily subject to sampling effects. The most sensitive genotypes are likely to be fully homozygous, because they are less frequent in the F_2_. Click here for file

Additional file 3 Effect of the missing genotypes estimation on the overall dynamics. The table summaries the four missing genotypes and their estimates following three different methods: taking the average of the population (624 g), estimating from the mean of the neighbor genotypes and estimating from the regressions of Figure 2. The figure presents the resulting dynamics, with the same parameters as Figure 3B. The differences are of the same order of magnitude as the stochastic differences between the repetitions, and the method to estimate the missing genotypes appears to have almost no influence on the results. Click here for file

Additional file 4 Impact of the simplification of the model. Simulations were simplified compared with the actual selection experiment. One of these simplifications was to consider an even and constant number of males and females selected each generation; the other was to consider that the population size was constant in both lines. Simulations show that the results remain consistent when these hypotheses are relaxed. Click here for file
