# Supplementary material for: Phenotypic evolution from genetic polymorphisms in a radial network architecture
Source: BMC Biol. 2007 Nov 14;5:50. doi: 10.1186/1741-7007-5-50 (PMC2194667; doi:10.1186/1741-7007-5-50)

SUPPLEMENTARY FIGURE 1

Effect of the presence of extra low-effect QTLs on the expected dynamics (initial frequency f(H) = 3/7). Part A presents the average selection response of the network itself, and of the network plus 6 small effect independent loci (-30g for LL genotype, +30g for HH genotype). The curves are scaled (same initial and final states) to be compared easily. Increasing the number of small additive QTLs clearly leads to a more linear response. B: comparison between the expected selection response, with or without additional QTLs (dotted lines) and the observed selection response (plain line). The observed response is the same as presented in Fig.1 (dynamics of body weight of males in the high line), but was corrected for sex effect (males being 25% heavier than females) and environmental effects (the average of the F_2 population being ~ 150g lower than expected from (i) the "base" population, and (ii) the average between high and low lines, this difference could not being explained by known dominance or epistatic effects).

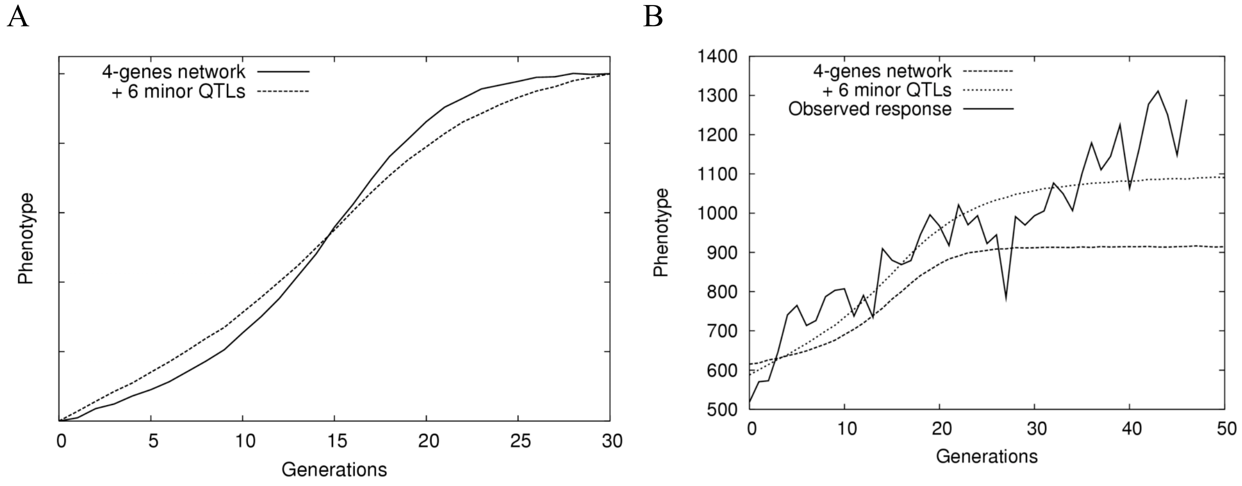

Supplement: Additional file 1 — Effect of the presence of extra low-effect QTLs on the expected dynamics. The average selection response of the network itself is compared with that of the network plus six small-effect independent loci. Increasing the number of small additive QTLs clearly leads to a more linear response that fits better with the experimental data. [file 1741-7007-5-50-S1.pdf]
