# Supplementary material for: Phenotypic evolution from genetic polymorphisms in a radial network architecture
Source: BMC Biol. 2007 Nov 14;5:50. doi: 10.1186/1741-7007-5-50 (PMC2194667; doi:10.1186/1741-7007-5-50)

SUPPLEMENTARY FIGURE 2

Sampling effect. The genotype-phenotype map was evaluated from a finite F_2 population, and the estimates of genotypic values are necessarily subject to sampling effects. The most sensitive genotypes are likely to be the fully homozogous ones, because they are less frequent in the F_2.

To check if this sampling effect may have an impact on the results of our simulations, we computed "noised" F_2 populations, similar to the real one, but in which the phenotype of the 538 individuals is changed (the phenotype P_i of individual i will be replaced by $P_i' = P_i + \varepsilon_i$, where ε_i is sampled in a Gaussian distribution of variance σ^2_R, *i.e.* the estimate of the residual variance in the F_2). The genotype-phenotype map was then computed in the same way as for the real dataset, and the figures below represent the results of the corresponding simulations for 5 independent sampling repetitions (same parameters as Fig 4).

The shape of the response to selection is really similar for each of the sampled genotype-phenotype map (A), but their amplitude (and therefore the ending point) is highly sensitive to the value of the "best" genotype (H allele fixed at each locus). However, the evolutionary dynamics of the system is not disturbed by sampling effects, *e.g.* the evolution of allelic frequencies in the course of time (B) remain very similar to the results presented in Fig 4, and the conclusions are not affected by potential sampling errors in the original dataset.

A

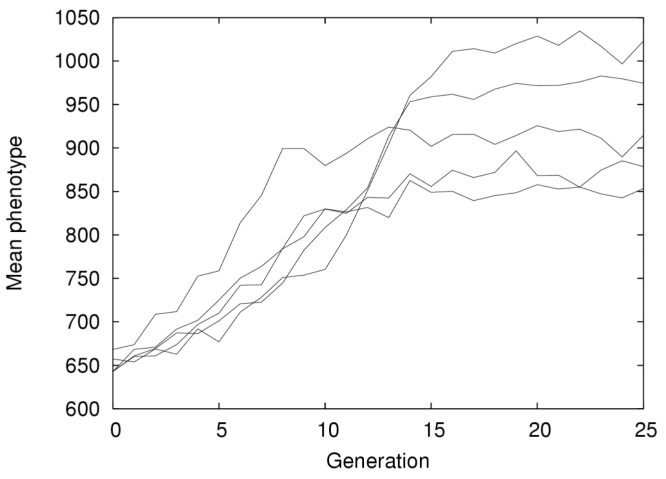

B

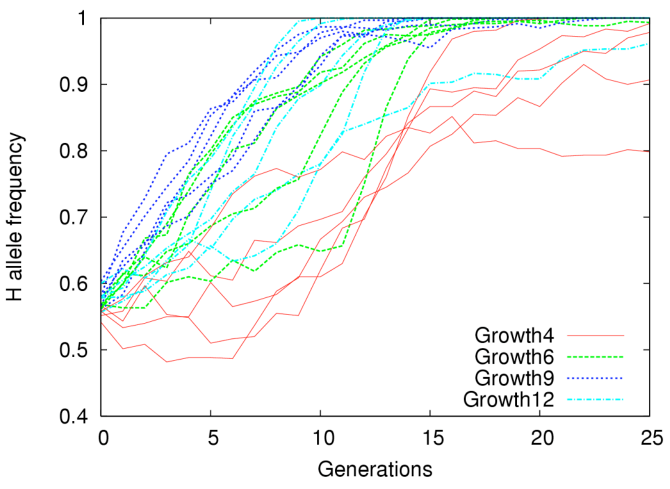

Supplement: Additional file 2 — Impact of the sampling effect. The genotype-phenotype map was evaluated from a finite F2 population and the estimates of genotypic values are necessarily subject to sampling effects. The most sensitive genotypes are likely to be fully homozygous, because they are less frequent in the F2. [file 1741-7007-5-50-S2.pdf]
