# Supplementary material for: Phenotypic evolution from genetic polymorphisms in a radial network architecture
Source: BMC Biol. 2007 Nov 14;5:50. doi: 10.1186/1741-7007-5-50 (PMC2194667; doi:10.1186/1741-7007-5-50)

SUPPLEMENTARY FIGURE 3

Effect of the missing genotypes estimation on the overall dynamics. The table summaries the four missing genotypes and their estimates following three different methods: taking the average of the population (624g), estimating from the mean of the neighbor genotypes, and estimating from the regressions of Figure 2. The figure below presents the resulting dynamics, with the same parameters as Figure 3B. The differences are in the same order of magnitude as the stochastic differences between the repetitions, and the method to estimate the missing genotypes appears to have almost no influence on the results.

Growth4	*Growth6*	*Growth9*	*Growth12*	Average	Neighbors	Regression
HH	LL	HH	LL	624	605	630
LL	HH	HH	HH	624	620	730
LL	HH	LL	HH	624	609	560
LL	LL	LL	HH	624	606	590

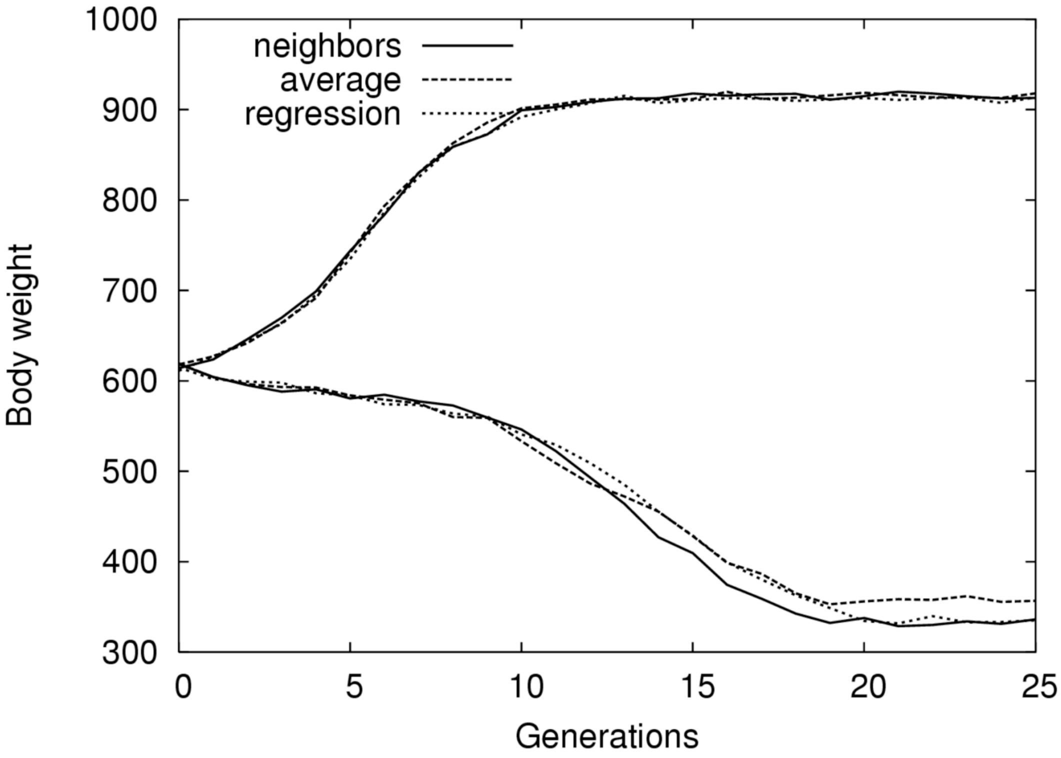

Supplement: Additional file 3 — Effect of the missing genotypes estimation on the overall dynamics. The table summaries the four missing genotypes and their estimates following three different methods: taking the average of the population (624 g), estimating from the mean of the neighbor genotypes and estimating from the regressions of Figure 2. The figure presents the resulting dynamics, with the same parameters as Figure 3B. The differences are of the same order of magnitude as the stochastic differences between the repetitions, and the method to estimate the missing genotypes appears to have almost no influence on the results. [file 1741-7007-5-50-S3.pdf]
