# Supplementary material for: Phenotypic evolution from genetic polymorphisms in a radial network architecture
Source: BMC Biol. 2007 Nov 14;5:50. doi: 10.1186/1741-7007-5-50 (PMC2194667; doi:10.1186/1741-7007-5-50)

SUPPLEMENTARY FIGURE 4

Simulations were simplified compared to the actual selection experiment. One of these simplifications was to consider an even and constant number of males and females selected each generation. The real selection procedure (described in the material and methods selection) has been simulated and compared to the simple case (20 males and 20 females selected each generation) in a 150 individuals population (Figure A below), and the average resulting dynamics appear to be almost identical (average of 10 simulations plotted).

A second approximation was to consider that the population size was constant in both lines. The actual populations used to fluctuate (see material and methods), and figure B below compare the average dynamics (10 simulations) when the population size is constant ($N_T = 300$), when it slightly fluctuates each generation (uniform distribution between 250 and 350 individuals), and when it largely fluctuates (between 200 and 400 individuals). Once again, differences appear to be very small.

A

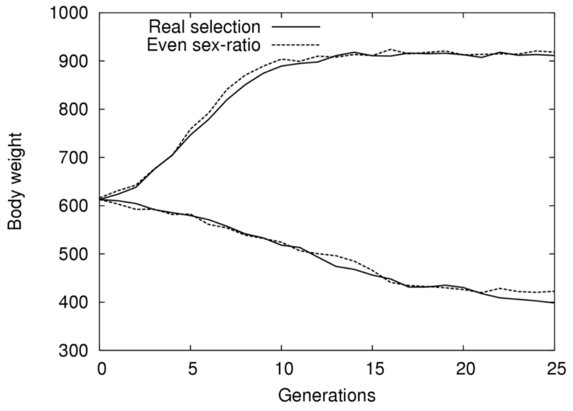

B

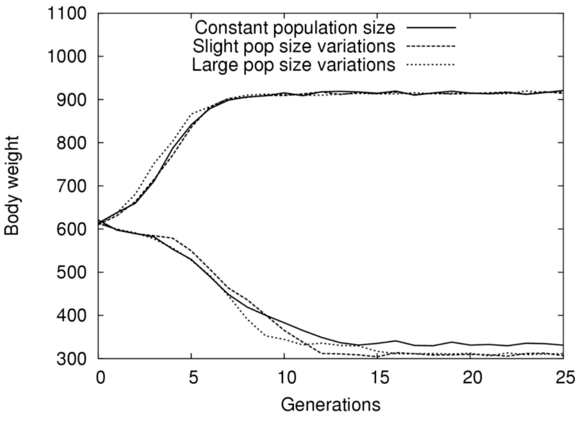

Supplement: Additional file 4 — Impact of the simplification of the model. Simulations were simplified compared with the actual selection experiment. One of these simplifications was to consider an even and constant number of males and females selected each generation; the other was to consider that the population size was constant in both lines. Simulations show that the results remain consistent when these hypotheses are relaxed. [file 1741-7007-5-50-S4.pdf]
